# The district hospital: A rare story of success and a way forward

**DOI:** 10.4102/phcfm.v17i1.4780

**Published:** 2025-01-16

**Authors:** Jason Fader, Tim Fader

**Affiliations:** 1 Department of Surgery, Faculty of Medicine, Hope Africa University, Bujumbura, Burundi; 2 Department of Medicine, Faculty of Medicine, Hope Africa University, Bujumbura, Burundi

**Keywords:** district hospital, health care systems, sustainability, first referral hospital, primary healthcare

## Abstract

The district hospital is a vital part of the healthcare system in sub-Saharan Africa. It is the key to enabling Primary Health Care to be done well, which in turn can allow for Universal Health Coverage to be achieved. There are numerous challenges at district hospitals making successful ones somewhat rare. One example of a successful district hospital is put forth in this article. In order to increase the number of successful district hospitals a means of sharing solutions to common problems needs to be created, which would ideally be an online repository of said solutions, available to any interested district hospital.

Universal Health Coverage is Target 3.8 of the Sustainability Development Goals (SDGs) adopted by the United Nations in 2015.^1^ This laudable goal is only possible through a robust Primary Health Care system and the district hospital is the lynchpin to making Primary Health Care work. The district hospital should be the workhorse of any robust healthcare system, having the ability to care for the majority of patients and serving as the hub for the vital community health programmes. Yet, despite this crucial role district hospitals remain an underutilised part of health systems in most every country in sub-Saharan Africa. The district hospital (or First Referral Hospital) in sub-Saharan Africa has had difficulty with its effectiveness and role in the larger health system for a long time. Mazhar et al.^2^ give a good synopsis of the history of the district hospital in sub-Saharan Africa and the lack of attention, which has been accorded to it despite its vital role. English et al.^3^ and McCord et al.^4^ give a good summary, in Disease Control Priorities Two and Three, respectively, of the essential role and value that the district hospital could and should have.

The Lancet Commission on Global Health^5^ elucidates that there are five billion people who lack access to safe surgical, anaesthesia, and obstetrical care around the world. In order to provide care to these five billion people, the district hospital itself must be healthy. We need to look at and treat the district hospital as a sort of patient in and of itself, using the principles of preventative care, good diagnosis and treatment protocols, and research for unresolved issues geared towards resolving the unique needs of the district hospital. Unless there is a focus on enriching and supporting the district hospital itself, comprehensive Primary Health Care, and therefore Universal Health Coverage, cannot be realised.

There is so much written about *what* the district hospital should do – whether it be the Alama-Ata Declaration, Millenium Development Goals (MDGs), SDGs, or other high-level mandates. Yet we would contend that these commendable mandates have largely failed to be implemented because of a lack of equal emphasis on *how* to put these policies into practice – the nuts and bolts of making a district hospital work. There is difference between having a good set of architectural plans for a house and actually knowing how to swing a hammer and lay the brick. For example, if a district hospital is to care for neonates, but has no functional incubator, it cannot provide that care or in the absence of oxygen a district hospital cannot treat patients with pneumonia. If the hospital is to provide Caesarean for obstructed labour but the needed suture is out of stock or the steriliser is out of commission, the Caesarean is impossible. Knowing *what* a hospital should do accomplishes very little if the hospital does not know *how* to do it. There are particularities and challenges to the context of the district hospital that call for strategies and solutions, which do not necessarily work in the developed world and so using policies, equipment, staffing ratios, and patient care algorithms designed for developed countries cannot be directly applied. Indeed, many district hospitals in sub-Saharan Africa function poorly and examples of successful growth are few, but there are certainly some exceptions. One of these is Kibuye Hope Hospital in Burundi.

## The history of Kibuye Hope Hospital

Kibuye Hope Hospital was started in 1946 by Free Methodist missionaries from the United States. It grew haphazardly over the ensuing decades because of civil wars. By 2013, it was an 80-bed district hospital with 86 employees. In that year 12 208 outpatients were seen, 1247 patients were admitted to the hospital, and there were 609 operations performed (the majority of which were c-sections). Around that time the hospital was acquired by and managed under Hope Africa University (located in the capital, 3 h away) to use as a rural clinical training site for their medical students. Furthermore, a team of six specialist physicians moved to the hospital in late 2013 to function as the core teaching faculty for these medical students. Over the ensuing 10 years the hospital has grown steadily in the areas of training, patient volume, capacity, and infrastructure. Noteworthy achievements include the training of over 300 medical students in addition to hundreds of nursing and ancillary medical personnel, a solar power system, which provides 96% of the hospital’s electricity, addition of an internship programme and 5-year surgical residency, with a Family Practice residency on the cusp of starting, pending ministry approval. The focus on medical education has helped tremendously with creating a culture of learning and improvement, as well as providing an increase in workforce. There has been an investment of around $9.2 million over the past 10 years, which has come primarily from private funding sources such as churches, individuals, and foundations. In 2023, the hospital had grown to 346 beds with statistics including 33 065 outpatient visits, 10 692 hospitalisations, and 3627 operations – 402%, 270%, 857%, and 596% increases, respectively. Throughout this time the hospital remained and still remains under Burundian leadership.

This remarkable growth was helped along by several factors, including adequate funding sources, a constant presence of long-term volunteer specialist staff, key partnerships, and a unified vision. In addition, there were many lessons learned in these past 10 years in terms of *how* to make the hospital work so it could fulfil its role as a district hospital and more. These lessons would likely be useful to the thousands of other district hospitals in sub-Saharan Africa who are facing similar challenges. Undoubtedly, there are also other district hospitals that have discovered solutions to problems that Kibuye Hope Hospital still struggles with. Kibuye Hope Hospital has now outgrown its role as a district hospital and now functions more as a referral, or university teaching hospital. Nevertheless, sharing these ideas, solutions, and persistent questions would prevent fellow hospitals from having to learn costly, time-consuming lessons and give them a bit of a roadmap for development. While not every district hospital will have the advantages of solid funding and volunteer specialist staff, having easily accessible solutions to improve the care and functioning of the hospital will enable interested hospitals to make significant strides.

## Growing the district hospital

There are five main areas that district hospitals need to consider when looking at functioning and development. They are:

- Understanding the district hospital’s role in the health system
- Administration and governance
- Infrastructure and equipment
- Training
- Patient care

**FIGURE 1:**
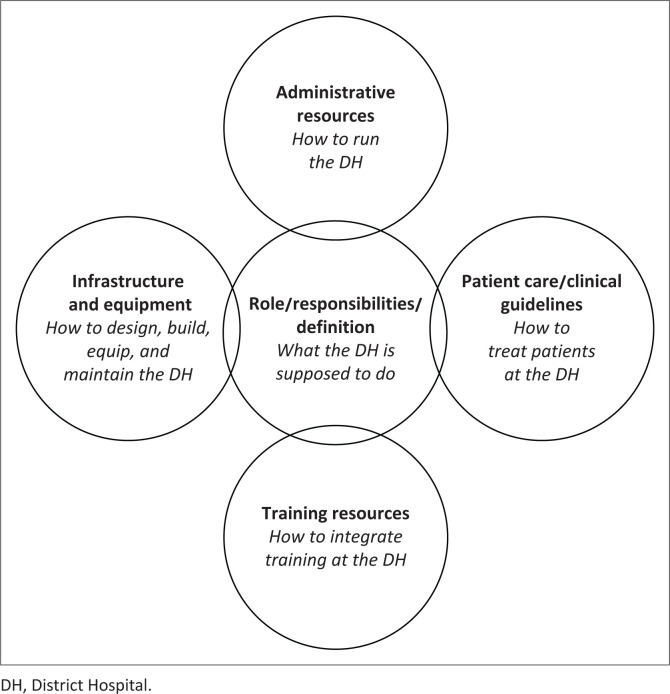
Areas of development in a district hospital.

Of these categories, there are just a few resources available, which have helped us in guiding the growth at Kibuye, namely in the areas of the hospital’s role and in patient care (Figure 1). The other three categories have a dearth of accessible information or resources geared towards helping district hospitals to grow and function well.

There needs to be a better way to develop and share successful strategies and solutions for the functioning and growth of the district hospital. We propose that an online repository of district hospital ideas, questions, and solutions be created. This would be in a similar format to Wikipedia, where there is a small editorial team receiving and editing contributions from a vast network of medical personnel, administrators, engineers, and public health workers. They would contribute ideas and solutions from their district hospital experience and there could also be a discussion board where problems are raised and solutions are proposed. Articles in this online repository would focus primarily on the *how* questions and could be written on topics such as ideal operating room layouts, managing a poor patient fund, recommended staffing ratios, how to build a low-cost labour bed, incorporating Family Practice residents into community health efforts, and guidelines on how to run a good Morbidity and Mortality conference. In addition, interested parties could convene biannually to present and discuss new ideas on the district hospital as a part of a Primary Health Care conference or as a stand-alone conference.

As it would be an online repository, it would easily be accessible to almost anyone, such that geographic and professional isolation would not prevent a biomed technician from learning how to build a low-cost wound vac or an administrator to have a solid sample contract or a surgeon to know how to make rush rods to repair long bone fractures.

While every district hospital has its unique peculiarities and challenges, there are certainly principles and solutions that would apply across the vast majority of these hospitals to enable them to be more effective and efficient. Providing a way for district hospitals to be healthy and thrive will allow them to be the enablers of Universal Health Coverage through Primary Health Care and provide, among other things, access to safe surgery and anaesthesia for those five billion people who currently suffer without it.

## References

[1] United Nations Department of Economic and Social Affairs - Sustainable Development [homepage on the internet]. No date [cited 2024 Sep 9]. Available from: https://sdgs.un.org/goals

[2] Mazhar RJ, Willows TM, Bhattarai S, Tinn C, Misago N, English M. First referral hospitals in low- and middle-income countries: The need for a renewed focus. Health Policy Plan. 2024;39:224–232. 10.1093/heapol/czad120 38386923 PMC11031140

[3] English M, Lanata CF, Ngugi I, Smith PC. The district hospital. In: Jamison DT, Breman JG, Measham AR, Alleyne G, Claeson M, Evans DB, Jha P, Mills A, Musgrove P, editors. Disease control priorities in developing countries. 2nd ed. New York: Oxford University Press, 2006; p. 1211–1228

[4] McCord C, Kruk ME, Mock CN, et al. Organization of essential services and the role of first-level hospitals. In: Debas HT, Donkor P, Gawande A, Jamison DT, Kruk ME, Mock CN, editors. Disease control priorities in developing countries. 3rd ed. Essential Surgery. New York: Oxford University Press, 2015; p. 213–2301.

[5] Meara JG, Leather AJM, Hagander L, et al. Global surgery 2030: Evidence and solutions for achieving health, welfare, and economic development. Lancet. 2015; 386(9993):569–624. 10.1016/s0140-6736(15)60160-x 25924834

